# The Book World of Medicine and Science

**Published:** 1893-05-06

**Authors:** 


### THE BOOK WORLD OF MEDICINE AND SCIENCE.

### APRIL QUARTERLIES.

IN the QUARTERLY REVIEW an article on the Life and Speeches of Sir Henry Maine discusses the complicated questions connected with the marriage laws in India. The intricacy of what the reviewer terms this interesting department of Indian sociology is hardly realised by those not familiarised with Indian ways by long residence in the Empire. Very remarkable contingencies are provided against in the divorce law, the necessity for which is shown by the following curious illustration: "A Hindu was converted to Christianity, and as a Christian married a Christian girl. He then reverted to Hinduism, and it seems to have been found out that he could be re-admitted to caste on certain conditions, the principal of which was, I am informed, that he should have his tongue pierced with a red-hot iron. He then proceeded to marry one or more Hindu wives, and was indicted under the penal code for bigamy. The High Court decided that, having relapsed into Hinduism, he re-acquired his rights of polygamy." Sir Henry Maine argued that while it would be inexpedient "to deny that a native of India may acquire a right to plurality of wives through the actual cautery of the tongue, yet we can and ought to relieve the Christian wife from the marriage bond." Mr. Aitken's book on Dr. Arbuthnot forms the subject of an essay, dwelling more on his political influence than on his professional achievements. His abilities were, indeed, turned in almost every direction rather than the advancement of his calling, though he stood *facile princeps* in his profession. He was said to be skilled in everything which related to science, was the first mathematician of his age, and an accomplished musician, art critic, and satirist. It is in these varied forms that his reputation survives rather than as the Court physician who failed in saving the unhappy little Heir-Apparent from the *régime* which led indirectly to his premature death. The Duke of Argyle's latest contribution to Economic Science already noticed in these columns, is discussed at length, and in Literary Discoveries in Egypt, much light is thrown on Egyptian customs in early Biblical times.

THE EDINBURGH REVIEW has a remarkable account of the French physician and naturalist of last century, Philibert Commerson. Recognised as one of first botanists of the age, he was the correspondent of Linnæus, and in the course of his widely extended travels collected a herbarium, afterwards deposited in the Jardin des Plantes, of incredible richness. In his collection were 5,000 plants, of which 3,000 species were new to science, forming 160 genera hitherto unknown to naturalists. His adventures in search of botanical specimens were singularly wild and romantic, but perhaps the most alarming was that which befel him at the commencement of his travels, when his dog went mad after falling into a swarm of bees, and infected him with rabies—"The commencement of the horror for drink, convulsive movements in the muscles of the throat, eyes, and face, an insupportable palpitation with pain and inflammation about the cicatrised wound, were phenomena too independent of the imagination to be mistakable. I then bethought myself of mercury, the one remedy against hydrophobia which can be trusted, but considering that I had not enough time to put in practice the precautionary

THE BOOK WORLD OF MEDICINE AND SCIENCE.—(*continued*).

measures preliminary to its external application, I had immediate recourse to such internal mercurial preparations as Aquila alba and mineral turbith mixed with the pulp of tamarinds in the manner of theriac. The alarming symptoms were suspended, but only for four or five weeks, after which they recommenced with even more violence. I again checked them by the same means, after which I added baths and frictions with Neapolitan ointment on the extremities. The ptyalism which appeared marked the epoch of my cure." After undergoing every description of risk and hardship, Commerson died at Mauritius in his forty-fourth year, some days before his spontaneous election to the Académie des Sciences. A sympathetic review of Proctor's "Old and New Astronomy" does good service in calling attention to the real services to science of one too often classed as a purely popular and superficial writer. A sketch of the history of Fontainebleau has many amusing bits of gossip extending from 1137 to 1870, and skims over the surface of French history pleasantly enough. "The Foreign Tours of Lady Mary Coke" deal with English Court gossip of the age of George II., and comprises also some well-touched pictures of foreign contemporary Courts.

THE BOOK WORLD OF MEDICINE AND SCIENCE—(*continued*).

variola, in others was like eczema and psoriasis and pityriasis-rubra. Out of 37 decided cases as many as six succumbed to the disease, including one case of suicide.

---

### NEW BOOKS.

London. By Walter Besant. (Chatto and Windus.)

No one has a better knowledge of the London of to-day, in its moral as well as its topographical aspects, than Mr. Besant. His books have opened up many unexplored regions to readers unacquainted even with the names of the localities described except through the pages of "Bradshaw." In the volume before us he applies his well-known methods (minus only the Besantian young lady) to a review of the City at successive periods of its history. Intensely interested himself in every detail of his subject, the author cannot fail to compel interest from his readers, and the value of this retrospect, gathered from original sources, of the social condition of Londoners, is independent even of its interest. Of the sanitary condition of London at various periods there are many details. The most remarkable fact to be noted in this connection is the continual tendency to retrograde movement in spite of spasmodic efforts at improvement. We are prone to think we have been growing steadily cleaner and healthier. It is well to be reminded that periods like the present of enthusiastic reform in such matters are apt to invite a reaction of dull apathy, succeeded by general backsliding. For instance, in the Saxon and Norman period we read, "At the corners of the streets are lay-stalls, where everything is flung to rot and putrefy, the streets are like our country lanes, narrow and muddy; public opinion is against shooting rubbish into the street, but it is done; the people walk gingerly among the heaps of offal and refuse." Six hundred years passed by. Plague after plague preached its terrible lesson and provoked short-lived efforts at reform. "Thirteen great pestilences fell upon the city between the years 1094 and 1625—in the last year 35,000 died. That is to say one plague happened about every forty years, so that there never was a time when a recent plague was not in the minds of men." Yet in 1754 a contemporary pamphlet, advocating reforms in the city, reveals a state of things in many respects entirely similar to that of Norman times. "For instance, the streets were not cleaned except in certain thoroughfares; at the back of the Royal Exchange was a scandalous accumulation of filth suffered to remain, and the posterns of the City gates were equally neglected and abused. The rubbish shot into the streets was not cleared away; think of the streets all discharging the duty of the dust-bin." A hundred years' immunity from the plague had been sufficient to slacken all sanitary precautions, for the spread of small-pox and infectious fevers had not yet been traced to these infringements of Nature's laws. And if sanitary science had reached a low ebb at the end of last century, what shall we say of medicine and surgery? "The citizen of the lower class never called in a physician unless he was in immediate danger; the herbalist physicked him, and the wise woman. And the doctor who wished to attract the confidence of citizens found a little stage management useful. He wore black, of course, with a huge wig; he carried a gold-headed cane, with a pomander box on the top; he kept

THE BOOK WORLD OF MEDICINE AND SCIENCE—(continued).

his hands always in a muff that they might be soft, warm to the touch, and delicate; he hung his consulting-room with looking-glasses, and littered it with vials; he had on the mantel-shelf a skull, and hanging to the wall the skeleton of a monkey; on his table stood a folio in Greek, and he preserved a Castilian gravity of countenance." Yet he frequently found himself out-run in the race for popularity by the bolder quack who advertised freely, drove about ostentatiously in a glass coach, and "cured by sympathy, by traction, by earth bathing, by sea bathing, by the quintessence of Bohea tea and cocoa-nuts distilled together, by drugs, and by potions."

ATLAS OF CLINICAL MEDICINE. By BYROM BRAMWELL, M.D., F.R.C.P.E., &c. (Edinburgh: A. and T. Constable, University Press.)

The second part of the second volume of Dr. Byrom Bramwell's gigantic undertaking is now issued. The subject of syphilis occupies the larger number of pages in this second part. The letterpress is well up to date, and is thoroughly practical and serviceable. But the illustrations, as in the preceding parts, constitute the unrivalled claims of this great work to the attention of the practitioner and the student. To say of these illustrations that they are accurate is to say little. To the careful observer they speak. We do not know what may have been the success of Dr. Byrom Bramwell's work from a financial point of view; but we are beginning to see the kind of monument it will build up for the author's reputation, and the unique services it will render to the busy practitioner, to whom a glance at a pictorial representation is often of more real service than whole volumes of poor and inadequate description. For it is to be remembered that the average medical writer is a sorry hand at descriptive presentation. Even the most skilful of descriptive writers might well tremble for his laurels if medicine were his only theme. But the camera, skilfully used, and the graver's art in competent hands, never fail to tell their story to those who have patient eyes. We wish some generous man of wealth, who has learned by experience to value the physician's skill, could send to every hard-worked and ill-paid doctor in the kingdom a completed copy of this noble "Atlas of Clinical Medicine." Certain it is that there is no medical library in the world but would be enriched by the presence of these volumes on its shelves.

HOW NATURE CURES: The Natural Food of Man. A Statement of the Principal Arguments against the Use of Bread, Cereals, Pulses, Potatoes, and all other Starch Foods." By EMMET DENSMORE, M.D. (London: Swan Sonnenschien and Co., Paternoster Square.)

The experienced reviewer gets in time to dread the handsome volume which has the outward and visible signs of having been published at the author's own expense. It is a sad commentary on human nature that it is the opinions of which the world stands least in need that an author is most ready to support by pecuniary means. Such books are not always wholly foolish; on the contrary, they often contain some theory, some germ of usefulness, of which an experienced journalist would say, "Put it in a pamphlet and we'll consider it." But the author, with money at his back, cannot be restrained from putting in all the irrelevant fancies that ever found a shelter in his brain. This is what Dr. Emmet Densmore has done. His theory of the natural food of man may be put in half a dozen words: "Minced

THE BOOK WORLD OF MEDICINE AND SCIENCE—*(continued)*.

beef, slowly stewed, and fruit as a second course." Why can he not say this and make an end ?

Because he was once a vegetarian ; and though now he has become carnivorous, he has filled his book with much that was obviously written in his vegetarian days. Thus, the story of a lady who got through a time of pain and danger with exceptional ease after a diet of roasted apples and sago with only the smallest quantity of fresh meat once a day, has no connection with the praise of the "Salisbury steak"—which is not a steak, but the stewed beef aforesaid—on which, without either bread or potatoes, the ideal man is to subsist hereafter. The one thread of connection between the various parts of the book is the author's hatred of bread. All cereals are bad, but wheat is the worst of all. Wholemeal bread does not escape his condemnation ; the aid it seems to give to digestion is deceptive ; it does but mechanically irritate the internal mucous membrane, and a dose of castor-oil were more wholesome fare. Bread, the so-called staff of life, is in truth "the staff of death" ; it is of the earth, earthy ; and earth to earth is the premature doom of him who touches it. True it is that though a poison it is, as the Scotchman said of whisky, a very slow poison ; and one may hang on, in spite of its pernicious effects, to three-score years and ten; but what is that to the patriarchal age we shall attain on "Salisbury steaks"? The Salisbury treatment has not been in existence for more than a very few years, but of its effect on longevity Dr. Densmore has not the faintest doubt. In fact, his trustful confidence reminds one of that Athenian philosopher who, hearing that a crow lived for three hundred years, bought a young crow in order to watch its growth and verify the hypothesis.

We do not insinuate for a moment that Dr. Densmore is wrong in the high estimate he has formed either of fruit as a food or of the "Salisbury diet." In certain cases of dyspepsia we have known a very simple meat diet, accompanied by severe restriction in the matter of starch foods to act admirably ; but mankind are not all dyspeptics, nor is all dyspepsia due to the same cause and to be cured by the same means. It is in this, we think, that Dr. Densmore, like most enthusiasts, errs. He sets down a treatment which has been successful with a limited number of invalids as "the natural food of man," and commands all mankind to live thereby; forgetting that all mankind need not be so limited, and that a healthy man can digest a far greater variety of substances than is here allowed. In fact a healthy man can, as a rule, live healthily on any food that is convenient to him. England has reared stalwart sons on a carnivorous diet, but we can hardly despise the result of Scotland's oatmeal porridge, or the offspring of Ireland's potatoes, "seasoned by a mythical condiment known as point."

Dr. Densmore forgets, too, that health, though largely affected by food, does not depend on that alone, but on occupations, exercise, amusement, and the like, and that the man who is always thinking of his food is, however carefully fed, as little likely to be in good physical health as he is to be in sound moral condition whose thoughts are always fixed upon the workings of his soul. To sum up our opinions of this book : the treatment which Dr. Densmore recommends is in itself sound, and would in many cases prove highly useful, but it is unnecessary to enforce it on healthy people, and to do so would weaken rather than strengthen the organs of digestion.

